# Bioinformatics analysis and identification of potential key genes and pathways in the pathogenesis of nonischemic cardiomyopathy

**DOI:** 10.1097/MD.0000000000037898

**Published:** 2024-04-26

**Authors:** Yan Jia, Rui-Ning Zhang, Yong-Jun Li, Bing-Yan Guo, Jian-Long Wang, Su-Yun Liu

**Affiliations:** a Department of Cardiology, The Second Hospital of Hebei Medical University, Shijiazhuang, China.

**Keywords:** bioinformatics analysis, differentially expressed genes, heart failure, nonischemic cardiomyopathy, protein–protein interaction network

## Abstract

Nonischemic cardiomyopathy (NICM) is a major cause of advanced heart failure, and the morbidity and mortality associated with NICM are serious medical problems. However, the etiology of NICM is complex and the related mechanisms involved in its pathogenesis remain unclear. The microarray datasets GSE1869 and GSE9128 retrieved from the Gene Expression Omnibus database were used to identify differentially expressed genes (DEGs) between NICM and normal samples. The co-expressed genes were identified using Venn diagrams. Kyoto Encyclopedia of Genes and Genomes pathway analyses and gene ontology enrichment were used to clarify biological functions and signaling pathways. Analysis of protein–protein interaction networks using Search Tool for the Retrieval of Interacting Genes/Proteins online to define the hub genes associated with NICM pathogenesis. A total of 297 DEGs were identified from GSE1869, 261 of which were upregulated genes and 36 were downregulated genes. A total of 360 DEGs were identified from GSE9128, 243 of which were upregulated genes and 117 were downregulated genes. In the 2 datasets, the screening identified 36 co-expressed DEGs. Kyoto Encyclopedia of Genes and Genomes pathway and gene ontology analysis showed that DEGs were mainly enriched in pantothenate and CoA biosynthesis, beta-alanine metabolism, kinetochore, G-protein beta/gamma-subunit complex, and other related pathways. The PPI network analysis revealed that DUSP6, EGR1, ZEB2, and XPO1 are the 4 hub genes of interest in the 2 datasets. Bioinformatics analysis of hub genes and key signaling pathways is an effective way to elucidate the mechanisms involved in the development of NICM. The results will facilitate further studies on the pathogenesis and therapeutic targets of NICM.

## 1. Introduction

Heart failure is an increasing world-wide problem and it is a major reason for the high morbidity and mortality of cardiovascular disease.^[1,2]^ With the advancement of therapeutic technology, survival in heart failure after diagnosis has been improved, but the mortality rate of patients is still high. There are many potential pathological reasons for heart failure, including hemodynamic pathology, infection, immune abnormalities, hereditary factors, toxins, metabolic disorders, and genetic mutations.^[3–5]^ Nonischemic cardiomyopathy (NICM) is a primary reason for advanced heart failure, which accounts for more than half of all heart transplants.^[6,7]^ Despite considerable improvements in the management of NICM, which can dramatically prolong patient survival and relieve patients’ symptoms,^[8]^ the mortality related to NICM remains a serious medical problem.^[9,10]^ Thus, it is necessary to find new critical targets to diagnose NICM early and improve the prognosis of the disease.

The research conducted in animal models and human studies can clarify the dysregulation of the key genes, proteins and important pathways, and can indicate the molecular mechanisms underlying disease initiation and progression. The pathogenesis of NICM is multiple, but they usually eventually lead to myocardial damage resulting in ventricular dysfunction and heart failure.^[10]^ Therefore, understanding the mechanisms and critical pathways in the pathogenesis is an important approach to prevent and diagnose NICM. With the development of microarray technology, the evaluation of gene changes has provided a new approach to investigate the regulation of cardiovascular disease.^[11–13]^ A study by investigators analyzing the gene expression in the hearts of 21 NICM patients and 10 ischemic cardiomyopathy identified common and unique genes expressed in NICM and ischemic cardiomyopathy.^[14]^ Therefore, the use of transcriptomic analysis can provide support for the treatment of NICM, and it can be used to diagnose NICM of different etiologies by analyzing changes in key gene expression and signaling pathways.

In this study, the microarray analysis data of heart samples from NICM patients were downloaded from the Gene Expression Omnibus (GEO) database, and bioinformatics analysis was used to explore the differentially expressed genes (DEGs), the changes in disease-related signaling pathways, and protein–protein interactions (PPI) in NICM. This can predict the relevant genes that play an essential role in NICM progression at the molecular level.

## 2. Materials and methods

### 2.1. Microarray data acquisition

The GEO database was used to obtain microarray data for NICM. GSE1869 and GSE9128 were downloaded from GEO. GSE1869 consists of 6 donor hearts and 21 NICM heart tissues, and GSE9128 contains heart tissues from 3 healthy donors and 4 NICM patients, respectively. Ethical approval was not required as a public database was used in this study and no patient data or privacy was involved.

### 2.2. Screening of differential expressed genes

The raw data from the GEO database were preprocessed and normalized by the Robust Multi-Array Average method based on the R software Affy package. The data were then subjected to DEGs analysis using the Limma package. *P* < .05 and |log2(FC)|>1 were set as the threshold, and the genes that met the criteria were considered DEGs. In addition, the heat map package of R software was used to generate the heat maps, highlighting the major regions of DEGs.

### 2.3. Gene ontology (GO) enrichment and Kyoto Encyclopedia of Genes and Genomes (KEGG) pathway analysis

Annotation and analyzing gene biological processes (BP), molecular functions (MF), and cellular components (CC) of DEGs were determined by using GO enrichment analysis. The route of the gene cluster and related biological functions was determined using KEGG pathway analysis. R cluster profile package was used to explore GO enrichment and KEGG pathway analysis with a cutoff criterion of adjusted *P* < .05.

### 2.4. Construction of the PPI network

The PPI network was analyzed using the Search Tool for the Retrieval of Interacting Genes/Proteins online to determine functional interactions between DEGs. PPI networks were visualized using Cytoscape software.

## 3. Results

### 3.1. Identification of DEGs in NICM samples compared with normal samples

**Figure 1.**
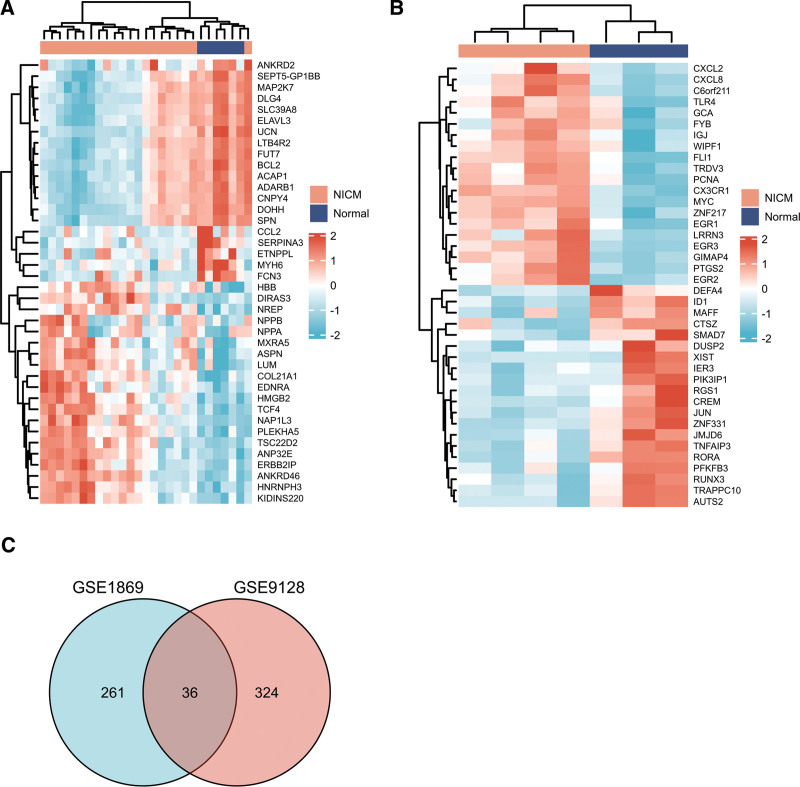
Heat map of the DEGs between NICM and donor heart. (A) Heat map of DEGs in GSE1869 dataset. (B) Heat map of DEGs in GSE9128 dataset. The gradient color change from blue to red indicates a change from downregulation to upregulation. (C) Venn diagram of DEGs. DEGs = differentially expressed genes.

A total of 297 differential genes were identified from GSE1869 dataset (261 upregulated, 36 downregulated), while 360 were identified in the GSE9128 dataset (243 upregulated, 117 downregulated). The heat map displayed the DEGs from GSE1869 and GSE9128 are shown in Figure 1A and B. Subsequently, we mapped the Venn diagram of the DEGs in the 2 datasets, and identified 36 co-expressed DEGs (Fig. 1C).

### 3.2. KEGG pathway analysis

**Figure 2.**
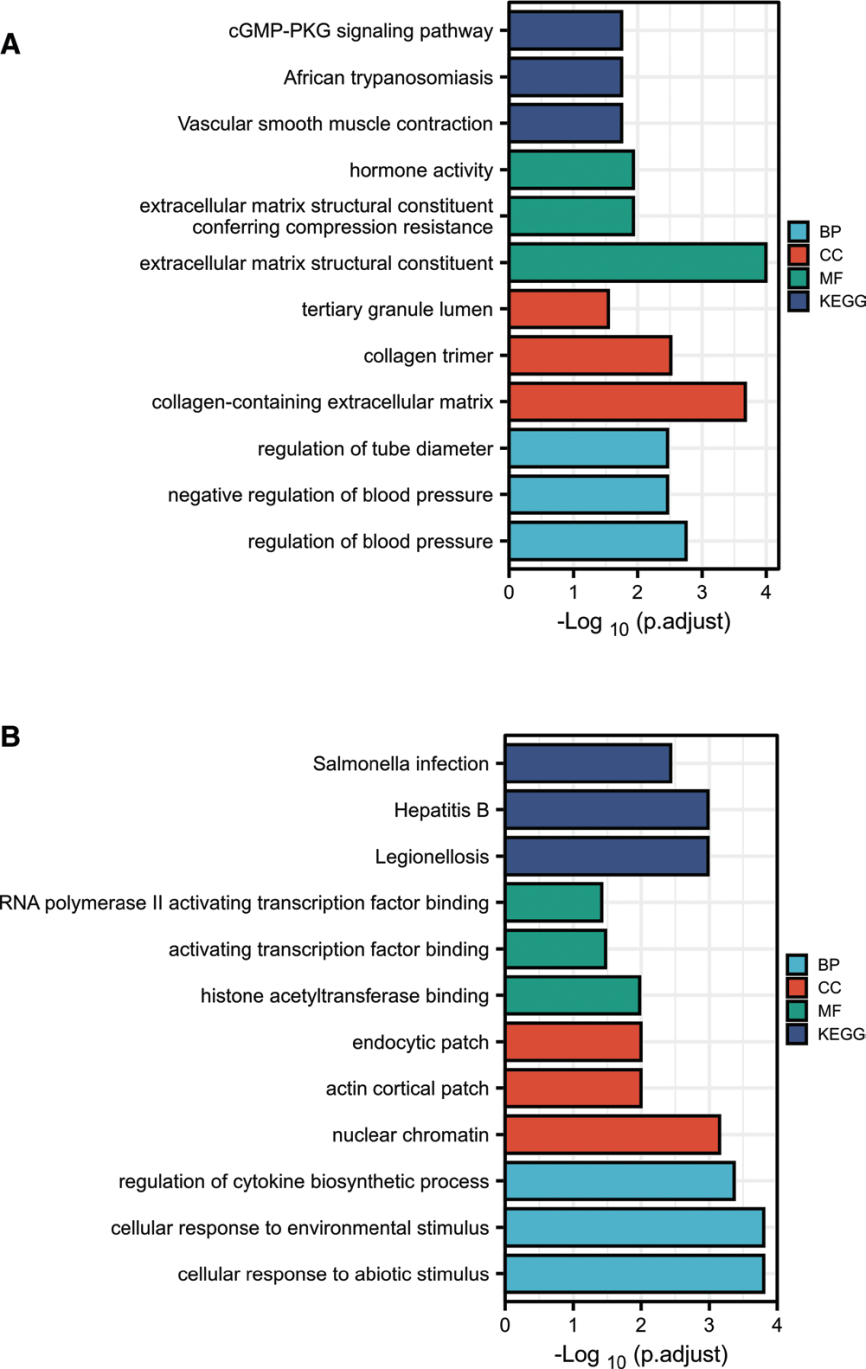
KEGG pathways and GO analysis of the DEGs. (A) The top 3 enriched KEGG pathways and GO terms in BP, CC, MF of GSE1869 dataset. (B) The top 3 enriched KEGG pathways and GO terms in BP, CC, MF of GSE9128 dataset. BP = biological process, CC = cellular component, DEGs = differentially expressed genes, GO = gene ontology, KEGG = Kyoto Encyclopedia of Genes and Genomes, MF = molecular function.

To get in more information about the noteworthy pathways of DEGs, a KEGG pathway analysis was exerted. The top 3 significant pathways of GSE1869 were mainly enriched in vascular smooth muscle contraction (*P* < .001), African trypanosomiasis (*P* < .01), cGMP-PKG signaling pathway (*P* < .01) (Fig. 2A). The top 3 significant pathways of GSE9128 were mainly enriched in legionellosis (*P* < .0001), hepatitis B (*P* < .0001), and salmonella infection (*P* < .0001) (Fig. 2B). Moreover, a total of 6 key pathways were found through the KEGG pathway analysis of co-DEGs (Table 1), which were primarily enriched in pantothenate and CoA biosynthesis (*P* < .05), beta-alanine metabolism (*P* < .05), pyrimidine metabolism (*P* < .05), drug metabolism-other enzymes (*P* < .05), GnRH signaling pathway (*P* < .05), and AGE-RAGE signaling pathway in diabetic complications (*P* < .05).

**Table 1:** KEGG pathway analysis of co-DEGs.

ID	Description	*P*-value	Gene ID
hsa00770	Pantothenate and CoA biosynthesis	.010362607	DPYD
hsa00410	Beta-alanine metabolism	.014778981	DPYD
hsa00240	Pyrimidine metabolism	.027939449	DPYD
hsa00983	Drug metabolism-other enzymes	.038564941	DPYD
hsa04912	GnRH signaling pathway	.045281109	EGR1
hsa04933	AGE-RAGE signaling pathway in diabetic complications	.048625967	EGR1

DEGs = differentially expressed genes, KEGG = Kyoto Encyclopedia of Genes and Genomes.

### 3.3. GO function enrichment analysis

GO analyses were conducted on GSE1869 and GSE9128 databases. The DEGs of GSE1869 dataset related to BP were primarily enriched in regulation of blood pressure (*P* < .0001), negative regulation of blood pressure (*P* < .0001), and regulation of tube diameter (*P* < .0001). Moreover, the enrichment analysis related to CC mainly were enriched in collagen-containing extracellular matrix (*P* < .0001), collagen trimer (*P* < .0001), and tertiary granule lumen (*P* < .01), whereas those relating to MF analysis were mostly enriched in extracellular matrix structural constituent (*P* < .0001), extracellular matrix structural constituent conferring compression resistance (*P* < .001), and hormone activity (*P* < .001) (Fig. 2A). The DEGs of GSE9128 dataset related to BP were primarily enriched incellular response to abiotic stimulus (*P* < .0001), cellular response to environmental stimulus (*P* < .0001), and regulation of cytokine biosynthetic process (*P* < .0001). The enrichment analysis related to CC showed that DEGs were involved in nuclear chromatin (*P* < .0001), actin cortical patch (*P* < .001), and endocytic patch (*P* < .001). In addition, the MF analysis was mainly enriched in histone acetyltransferase binding (*P* < .0001), activating transcription factor binding (*P* < .001), and RNA polymerase II activating transcription factor binding (*P* < .001) (Fig. 2B).

Moreover, GO analyses were performed on the detected co-expressed DEGs to examine their biological functions in detail. The enrichment analysis related to CC were primarily enriched in kinetochore (*P* < .01), G-protein beta/gamma-subunit complex (*P* < .01), and chromosome, centromeric region (*P* < .01). In addition, MF analysis showed that co-expressed DEGs were involved in DNA-binding transcription repressor activity, RNA polymerase II-specific (*P* < .001), DNA-binding transcription activator activity, RNA polymerase II-specific (*P* < .01), ubiquitin-like modifier activating enzyme activity (*P* < .01), nuclear export signal receptor activity (*P* < .01), and RAGE receptor binding (*P* < .01) (Table 2).

**Table 2:** GO enrichment analysis of co-DEGs.

ID	Description	*P*-value	Gene ID
GO:0000776	Kinetochore	.0040	XPO1, NSL1
GO:0031680	G-protein beta, gamma-subunit complex	.0078	GNG10
GO:0000775	Chromosome, centromeric region	.0080	XPO1, NSL1
GO:0001227	DNA-binding transcription repressor activity, RNA polymerase II-specific	.0007	ELK3, SP3, ZEB2
GO:0001228	DNA-binding transcription activator activity, RNA polymerase II-specific	.0036	EGR1, ELK3, ZEB2
GO:0008641	Ubiquitin-like modifier activating enzyme activity	.0073	UBA3
GO:0005049	Nuclear export signal receptor activity	.0081	XPO1
GO:0050786	RAGE receptor binding	.0081	HMGB2

DEGs = differentially expressed genes, GO = gene ontology, KEGG = Kyoto Encyclopedia of Genes and Genomes.

### 3.4. PPI network analysis

**Figure 3.**
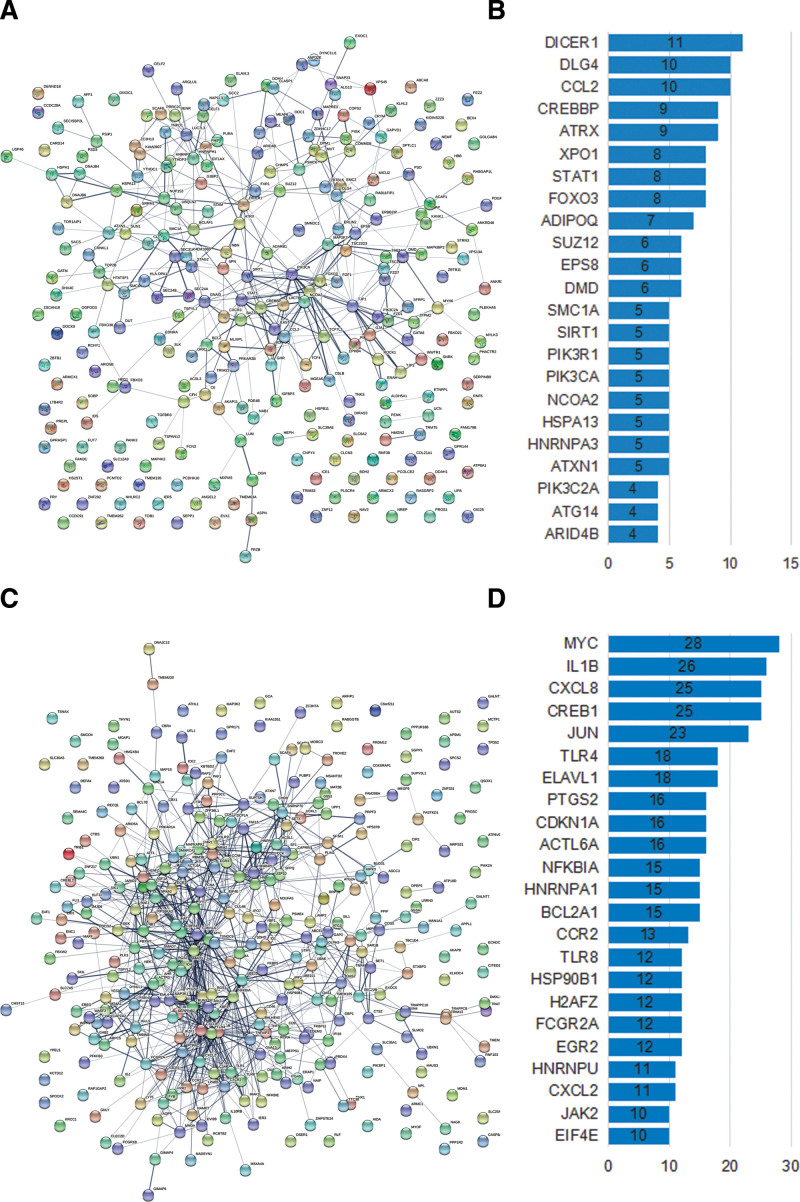
PPI network analysis. (A) PPI network of DEGs in GSE1869 dataset. (B) Histograms of core proteins of GSE1869 DEGs. The vertical and horizontal axes indicate the gene names and the number of gene connection, respectively, and the height represents the number of gene connection. (C) PPI network of DEGs in GSE9128 dataset. (D) Histograms of core proteins of GSE9128 DEGs. DEGs = differentially expressed genes, PPI = protein–protein interaction.

**Figure 4.**
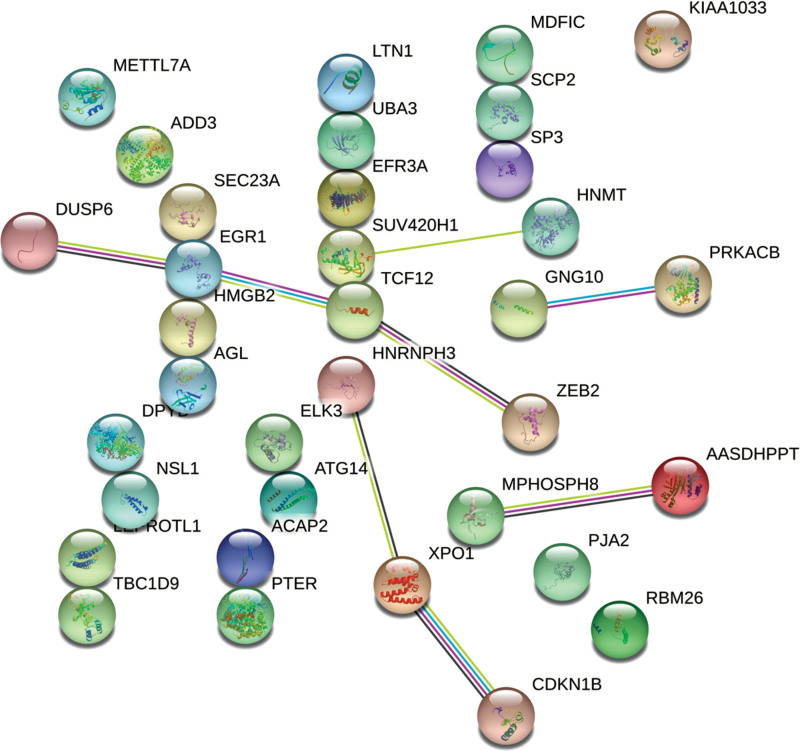
PPI network analysis of co-DEGs. DEGs = differentially expressed genes, PPI = protein–protein interaction.

Twenty-three major proteins were discovered in the PPI network analysis of GSE1869, with ribonuclease III (DICER1), discs large MAGUK scaffold protein 4 (DLG4), C-C motif chemokine ligand 2 (CCL2) as the most important, which connected 11, 10, and 10 nodes, respectively (Fig. 3A and B). Twenty-three major proteins were discovered in the PPI network analysis of GSE9128, with MYC proto-oncogene (MYC) connecting 28 nodes, interleukin 1 beta (IL1B) connecting 26 nodes, and C-X-C motif chemokine ligand 8 (CXCL8) connecting 25 nodes (Fig. 3C and D). Moreover, a PPI network analysis of DEGs was conducted with the Search Tool for the Retrieval of Interacting Genes/Proteins online database. As shown in Figure 4, dual specificity protein phosphatase 6 (DUSP6), early growth response protein 1 (EGR1), transcription factor 12 (TCF12), Zinc finger E-box-binding homeobox 2 (ZEB2), M-phase phosphoprotein 8 (MPHOSPH8), aminoadipate-semialdehyde dehydrogenase-phosphopantetheinyl transferase (AASDHPPT), heterogeneous nuclear ribonucleoprotein H3 (HNRNPH3), exportin-1 (XPO1), cyclin-dependent kinase inhibitor 1B (CDKN1B), G-protein subunit gamma 10 (GNG10), cAMP-dependent protein kinase catalytic subunit beta (PRKACB), lysine methyltransferase 5B (SUV420H1), and histamine N-methyltransferase (HNMT) are interconnected among the 36 co-DEGs, while the remaining proteins had no significant influencing characteristics.

## 4. Discussion

NICM is a widely prevalent disease characterized by multiple reasons for myocardial dysfunction. Although the underlying causes are various, NICM results in ventricular dysfunction.^[3,15,16]^ The morbidity and mortality of NICM remains high despite the availability of new therapeutic approaches, such as heart transplantation and cell therapy.^[6,8]^ Bioinformatics analysis provides useful information and assistance to identify the role of relevant genes, proteins, and signaling pathways in the development of disease.^[17,18]^ In this study, 36 co-expressed DEGs were identified in 2 datasets (GSE1869 and GSE9128). GO enrichment and KEGG pathway analyses were performed, and PPI networks were constructed to further analyze the molecular processes involved in NICM progression. Our results might facilitate an effective understanding of the molecular mechanisms involved in the development of NICM, and this could potentially identify therapeutic targets for NICM.

PPI network and key module analysis identified 4 related genes involved in NICM. DUSP6 is a member of the MAPK phosphatase family, which is highly specific to ERK1/2. When the myocardium was stretched DUSP6 expression was significantly increased through p38-MAPK pathway.^[19]^ In addition, Dusp6 was associated with cardiac repair, and inhibition of Dusp6 function could promote cardiac regeneration. Some evidence suggested that when Dusp6 inactivated it could increase cardiomyocyte proliferation, coronary angiogenesis and reduce fibrosis after ventriculotomy.^[20]^ Other studies have shown that DUSP6 regulates aortic and venous intercellular adhesion molecule-1 expression and is involved in the regulation of vascular endothelial inflammation.^[21,22]^

EGR1 played an important role in myocardial ischemia/reperfusion injury.^[23]^ EGR1 participated in the regulation of inflammation and fibrosis in postinfarcted hearts.^[24]^ Oxidative stress-induced EGR1 was involved in cardiac infarction, and it was able to effectively suppress ventricular arrhythmias in postinfarcted hearts by regulating EGR1 expression, which provided a new therapeutic strategy for ischemic arrhythmias in the clinic.^[25]^

ZEB2 regulated the pathogenesis of coronary artery disease by modulating metabolism and lipid function.^[26]^ It was found that ZEB2 was involved in regulating the occurrence of heart failure through direct binding to miR-215-5p, and this pathway provides new ideas for the treatment of heart failure.^[27]^

XPO1, a nucleocytoplasmic transport-related genes, had a potent relationship with left ventricular function parameters and could distinguish differentiation at the transcriptome level between dilated cardiomyopathy and ischemic cardiomyopathy and provide a basis for the therapeutic selection.^[28]^ In ischemic cardiomyopathy patients, XPO1 was high expressed and inversely correlated with left ventricular function. Knockdown of XPO1 expression attenuated cardiac dysfunction and remodeling in animal models after myocardial infarction.^[29]^

## 5. Conclusion

In this study, we found DEGs and related signaling pathways through a comprehensive bioinformatics analysis, and searched for potential biomarkers and predicted the progression of NICM by constructing a PPI network and identifying key modules. Pantothenate and CoA biosynthesis, beta-alanine metabolism, pyrimidine metabolism, drug metabolism-other enzymes, GnRH signaling pathway, and AGE-RAGE signaling pathway in diabetic complications might be essential signaling pathways in the pathogenesis of NICM. We discovered 4 genes of interest (DUSP6, EGR1, ZEB2, and XPO1) that might be used as diagnostic and prognostic indicators of NICM. Thus, our findings provided a potential rationale for understanding the etiology and mechanisms of NICM and the targets of the clinical treatment. As with most bioinformatic analysis studies of human diseases, this study has some limitations and further in vitro and in vivo studies will be conducted to confirm the role of relevant genes and signaling pathways in NICM.

## Author contributions

**Conceptualization:** Yan Jia, Su-Yun Liu.

**Data curation:** Yan Jia, Rui-Ning Zhang, Yong-Jun Li, Bing-Yan Guo.

**Formal analysis:** Yan Jia.

**Methodology:** Rui-Ning Zhang, Yong-Jun Li, Jian-Long Wang.

**Supervision:** Su-Yun Liu.

**Writing – original draft:** Yan Jia.

**Writing – review & editing:** Yan Jia.
